# Progressive increase in central nervous system immune activation in untreated primary HIV-1 infection

**DOI:** 10.1186/s12974-014-0199-y

**Published:** 2014-12-03

**Authors:** Joome Suh, Elizabeth Sinclair, Julia Peterson, Evelyn Lee, Tassos C Kyriakides, Fang-yong Li, Lars Hagberg, Dietmar Fuchs, Richard W Price, Magnus Gisslen, Serena Spudich

**Affiliations:** Yale School of Medicine, 367 Cedar Street, New Haven, CT 06510 USA; Department of Medicine, UCSF School of Medicine, 1001 Potrero Avenue, SFGH 100, San Francisco, CA 94143 USA; Department of Neurology, UCSF School of Medicine, 1001 Potrero Avenue, SFGH 1, San Francisco, CA 94110 USA; Yale Center for Analytical Sciences, Yale University, 300 George Street, Suite 555, New Haven, CT 06520 USA; Department of Infectious Diseases, University of Gothenburg, Journalvägen 10, 416 85 Gothenburg, Sweden; Division of Biological Chemistry, Innsbruck Medical University, Innrain 80, 6020 Innsbruck, Austria; Department of Neurology, Yale School of Medicine, 300 George Street, Room 8300c, New Haven, CT 06510 USA

**Keywords:** HIV, Central Nervous System Viral Infections, Inflammation, Neopterin, Activated T-cells, Immune Activation

## Abstract

**Background:**

Central nervous system (CNS) inflammation is a mediator of brain injury in HIV infection. To study the natural course of CNS inflammation in the early phase of infection, we analyzed longitudinal levels of soluble and cellular markers of inflammation in cerebrospinal fluid (CSF) and blood, beginning with primary HIV-1 infection (PHI).

**Methods:**

Antiretroviral-naïve subjects identified as having PHI (less than one year since HIV transmission) participated in phlebotomy and lumbar puncture at baseline and at variable intervals thereafter. Mixed-effects models were used to analyze longitudinal levels of CSF neopterin and percentages of activated cluster of differentiation (CD)4+ and CD8+ T-cells (co-expressing CD38 and human leukocyte antigen**-**D-related **(**HLA-DR)) in blood and CSF.

**Results:**

A total of 81 subjects were enrolled at an average of 100 days after HIV transmission and had an average follow-up period of 321 days, with the number of visits ranging from one to 13. At baseline, the majority of subjects had CSF neopterin concentrations above the upper limit of normal. The baseline concentration was associated with the longitudinal trajectory of CSF neopterin. In subjects with baseline levels of less than 21 nmol/L, a cutoff value obtained from a mixed-effects model, CSF neopterin increased by 2.9% per 10 weeks (n = 33; *P* <0.001), whereas it decreased by 6.7% in subjects with baseline levels of more than 21 nmol/L (n = 11; *P* = 0.001). In a subset with available flow cytometry data (n = 42), the percentages of activated CD4+ and CD8+ T-cells in CSF increased by 0.8 (*P* <0.001) and 0.73 (*P* = 0.02) per 10 weeks, respectively.

**Conclusions:**

Neopterin levels and the percentages of activated CD4+ and CD8+ T-cells in CSF progressively increase in most subjects without treatment during early HIV-1 infection, suggesting an accrual of intrathecal inflammation, a major contributor to neuropathology in HIV infection.

## Background

With the increasing availability of combination antiretroviral therapy (cART) the prevalence of HIV-associated dementia (HAD, the most severe form of HIV-associated neurocognitive disorder (HAND)) decreased from between 10 and 15% to about 2% in the HIV-1-infected population in the United States [1]. However, milder forms of HAND remain unchanged at a prevalence of between 40 and 50% and are associated with decreased quality of life, poor treatment adherence, and higher mortality risk [2–4]. While poor viral suppression is a contributor to this high prevalence, neurocognitive impairment is also observed in individuals with adequate cART adherence and suppressed viral loads [5,6] for reasons that are unclear. Studies have shown that inflammation, a mediator of central nervous system (CNS) injury [7], persists at a low level for many years despite cART [8–10], possibly causing continual damage to the CNS.

In exploring explanations for persistent CNS injury, some studies have focused on the impact of early infection prior to initiation of cART, since it is known that HIV enters the CNS and induces immune activation soon after transmission [11,12]. Emerging evidence from studies examining cerebrospinal fluid (CSF) and neuroimaging biomarkers in primary HIV infection (PHI, defined as within the first year of transmission) suggests that neuronal injury and dysfunction are already occurring in this stage [13–15]. These studies highlight the potential importance of the early stage of infection in HIV neuropathogenesis. Although cross-sectional studies have provided snapshots of processes occurring in the CNS during PHI [16], longitudinal processes occurring at this stage of infection in ART-naïve subjects have not been described.

In this study, we sought to examine the natural course of CNS inflammation starting in PHI. We hypothesized that CNS immune activation stimulated during initial infection would persist, and perhaps intensify, prior to initiation of ART, establishing a progressive inflammatory environment in the CNS. In a cohort of ART-naïve PHI subjects, we analyzed blood and CSF for longitudinal levels of soluble and cellular markers of inflammation. Neopterin was examined as a soluble surrogate biomarker of inflammation, and cluster of differentiation (CD)38 and human leukocyte antigen-D-related (HLA-DR) expressed together on CD4+ and CD8+ T-cells were examined as cellular markers of immune activation.

## Methods

### Study participants

ART-naïve HIV-1-infected subjects within one year of transmission were recruited in Gothenburg, Sweden (n = 17) and San Francisco, United States (n = 64). As described previously [16], PHI in the participants was confirmed by a combination of seroconversion, nucleic acid testing, or less-sensitive enzyme-linked immunosorbent assay (ELISA) testing, according to the serologic testing algorithm for recent HIV seroconversion (STARHS) methods [17]. The duration since transmission was estimated by considering the date at onset of seroconversion symptoms as 14 days post-transmission [18]. For participants without seroconversion symptoms (n = 16), we estimated the date of transmission as halfway between the most recent negative and positive HIV tests [19]. Protocols were approved by the University of California San Francisco Committee on Human Research (H9133-26278) and the Research Ethics Committee of the University of Gothenburg (Ö588-01). Informed consent was obtained from all participants prior to enrollment.

### Data collection

Longitudinal paired samples of CSF and blood were obtained prior to cART. In San Francisco, visits were scheduled at baseline (earliest enrollment after initial transmission), six weeks, and every subsequent six months from baseline, while in Gothenburg, study intervals were variable. Opportunistic infections and major neurological diseases such as stroke, multiple sclerosis, and seizure disorders were ruled out at baseline. Blood samples were analyzed for HIV RNA, albumin, CD4+ lymphocytes and CD8+ lymphocytes, and CSF samples were analyzed for HIV RNA, albumin, and white blood cell (WBC) count, as previously described [16]. Concentrations of CSF and blood neopterin were measured in previously frozen samples in the laboratory of Dr Fuchs by commercial immunoassays (BRAHMS Aktiengesellschaft, Hennigsdorf, Germany). A subset of fresh whole blood and CSF samples obtained in San Francisco (n = 42) was further analyzed by multiparameter flow cytometry as previously described [20,21] using FACSDiva (BD Biosciences, San Jose, USA) to measure the percentage of CD4+ and CD8+ cells with dual positivity for activation markers CD38 and HLA-DR. Data was analyzed with FlowJo (TreeStar, Ashland, Oregon, United States).

### Statistical analysis

All statistical analyses were performed using SPSS version 19, IBM Corp., Armonk, USA. All descriptive statistics are presented as median and interquartile range (IQR) for continuous variables, and as frequency (%) for categorical variables. Fischer’s exact test, the Mann-Whitney U test, and Spearman’s rank correlation coefficient were used where appropriate.

To analyze the longitudinal trajectory of CSF neopterin, we used mixed-effects models, which can accommodate differences among subjects in the total number of visits and intervals between visits. CSF neopterin concentrations were log-transformed to approximate a normal distribution before formal modeling. The models included fixed effects of baseline CSF neopterin and days post-transmission, and the random effect of intercept. The quadratic time effect was excluded from the final model because of statistical non-significance (*P* = 0.31). We also included an interaction term (days post-transmission × baseline CSF neopterin) as a fixed effect to examine if the trajectory of CSF neopterin over days post-transmission depended on the baseline level. Since baseline CSF neopterin was used as a predictor, only CSF neopterin values from subsequent visits were used as observations of the dependent variable.

This model yielded a regression coefficient for slope (change in CSF neopterin per day post-transmission) equal to 0.000294 - 1.397E-5 × baseline CSF neopterin, suggesting that the slope of CSF neopterin trajectory was dependent on baseline CSF neopterin. Based on this regression coefficient, baseline CSF neopterin values greater than 21 nmol/L yielded a negative slope, whereas baseline CSF neopterin values less than 21 nmol/L yielded a positive slope. Therefore, a baseline CSF neopterin level of 21 nmol/L was a cutoff value that determined the increasing versus decreasing trajectory of CSF neopterin. These results were confirmed by another model that used baseline CSF neopterin as a dichotomous variable (above or below 21 nmol/L). Although only the group with baseline CSF neopterin below 21 nmol/L included subjects with visits beyond 150 weeks, sensitivity analysis constraining the time axis to 150 weeks yielded the same trajectories as above. Therefore the full analysis, which included these visits, is presented in this paper.

Similarly, we used mixed-effects models to examine trends in the proportions of CD4+ and CD8+ cells co-expressing CD38 and HLA-DR in blood and CSF. In each of the four models, days post-transmission was a fixed effect, intercept was a random effect, and the percentage of cells co-expressing CD38 and HLA-DR was the outcome variable.

## Results

### Study participant characteristics

The characteristics of participants are shown in Table 1. Subjects were mostly male, had a median age of 36 years (IQR: 28 to 45), and were at a median of 100 days post-transmission at baseline. Although the median blood CD4+ T-cell count was 552 cells/μL (IQR: 389 to 720), one individual had an extremely low baseline CD4+ T-cell count of 111 cells/μL. The total number of visits ranged from 1 to 13, and the median duration of follow-up was 321 days.

**Table 1:** **Baseline demographic and laboratory features of subjects and follow-up duration**

**Baseline feature/lab value**	**Median (interquartile range)**
Total number of subjects	81
Male (%)	77 (95.1)
Age (years)	36 (28 to 45)
Duration since transmission at baseline (days)	100 (54 to 153)
Blood CD4+ T-cells (cells/μL)	552 (389 to 720)
Plasma HIV RNA (log10 copies/mL)	4.56 (3.97 to 5.18)
CSF HIV RNA (log10 copies/mL)	2.77 (1.80 to 3.47)
Plasma neopterin (nmol/L)	15.1 (9.4 to 21.6)
CSF neopterin (nmol/L)	10.2 (6.6 to 21.3)
% Blood CD4 + CD38 + HLA-DR + ^a^	7.5 (6.2 to 11.6)
% Blood CD8 + CD38 + HLA-DR + ^a^	55.3 (47.4 to 68.1)
% CSF CD4 + CD38 + HLA-DR + ^a^	14.2 (8.5 to 22.2)
% CSF CD8 + CD38 + HLA-DR + ^a^	70.9 (60.9 to 80.6)
Total number of visits	2 (1 to 3)
Duration of follow-up (days)	321 (105 to 727)

^a^n = 42 for flow cytometry analysis. CD, cluster of differentiation; CSF, cerebrospinal fluid; HLA-DR, human leukocyte antigen-D-related.

### Baseline measures of immune activation

Consistent with a previous report on a subset of this cohort [16], the median CSF neopterin concentration at baseline was 10.2 nmol/L (IQR: 6.6 to 21.3), with 81% of subjects having levels above 5.8 nmol/L, the upper limit of normal (ULN) [22]. Plasma neopterin at baseline was also elevated at 15.1 nmol/L (IQR: 9.4 to 21.6, ULN: 8.7 nmol/L [23]). Percentages of activated CD4+ and CD8+ cells in both blood and CSF were also elevated compared to published values in HIV-uninfected subjects obtained using similar methods (Table 1) [20]. Plasma neopterin correlated positively with the percentage of activated CD4+ cells (r_s_ = 0.38, *P* = 0.02) and CD8+ cells (r_s_ = 0.40, *P* = 0.02) in blood, and the percentage of activated CD8+ cells in CSF (r_s_ = 0.41, *P* = 0.01). CSF neopterin correlated moderately with the percentage of activated CD4+ cells (r_s_ = 0.36, *P* = 0.03) and CD8+ cells (r_s_ = 0.37, *P* = 0.03) in blood and strongly correlated with the percentage of activated CD8+ cells in CSF (r_s_ = 0.63, *P* <0.001).

### Post-transmission trajectory of cerebrospinal fluid neopterin

We next applied linear mixed-effects models to examine longitudinal changes in intrathecal immune activation prior to ART. A total of 44 subjects with a baseline CSF neopterin value and at least one follow-up value contributed to the analysis. Two significant trends were observed and depended on the baseline CSF neopterin concentration (interaction effect, *P* <0.001) being above or below 21 nmol/L, a cutoff value obtained from a mixed-effects model (see Methods). In participants with baseline CSF neopterin concentrations below 21 nmol/L, henceforth referred to as the ‘low group’ (n = 33), CSF neopterin levels increased over time, and a lower baseline value was associated with a steeper increase. This group included eight subjects with baseline CSF neopterin concentrations within the normal range. In contrast, CSF neopterin decreased in the minority of participants with baseline CSF neopterin concentrations above 21 nmol/L, henceforth referred to as the ‘high group’ (n = 11). Within this group, a higher baseline value was associated with a faster decline.

**Figure 1:**
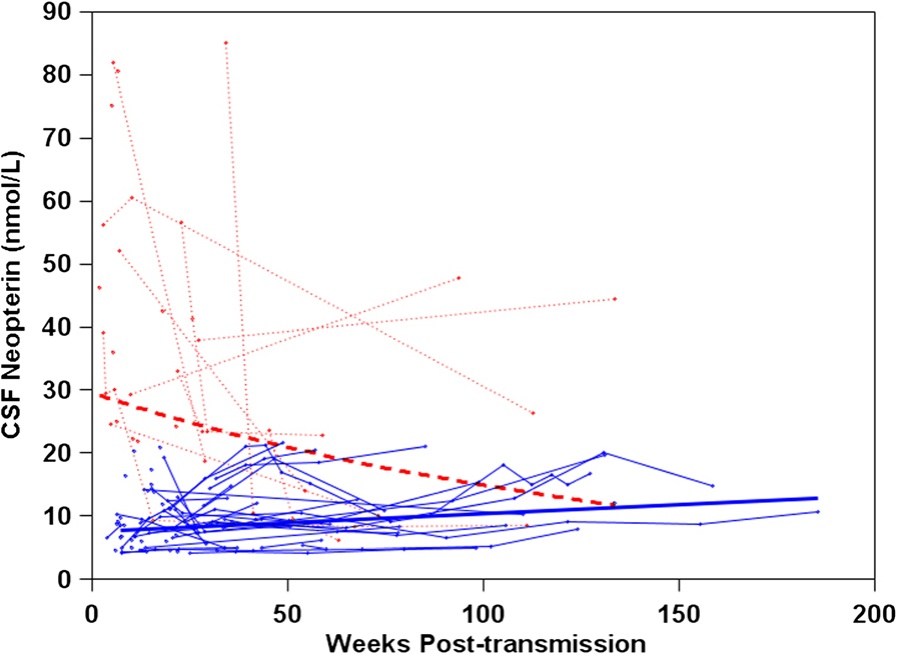
**Trajectories of cerebrospinal fluid (CSF) neopterin in early HIV-1 infection.** Thin lines represent the trajectories of individual subjects and bold lines represent the average slopes of high (red) and low (blue) groups (see text).

To confirm the observation of 21 nmol/L as a cutoff value, we ran a similar mixed-effects model with baseline CSF neopterin as a categorical variable. This model yielded the average change in CSF neopterin level over time separately for the low and high groups, and showed that they were significantly different as evidenced by the group-by-time interaction (*P* <0.001). As shown in Figure 1, CSF neopterin concentration increased in the low group by approximately 2.9% per 10 weeks (*P* <0.001), and decreased in the smaller high group by approximately 6.7% per 10 weeks (*P* = 0.001).

### Correlations with high and low baseline cerebrospinal fluid neopterin levels

When the groups with baseline CSF neopterin concentrations below and above 21 nmol/L were compared on baseline parameters (Table 2), significant differences were not found in age or days post-transmission. However, the high group was found to have a lower CD4+ cell count in blood (*P* <0.001), higher neopterin levels in plasma (*P* = 0.006) and CSF (*P* <0.001), and HIV RNA levels in both plasma (*P* = 0.02) and CSF (*P* = 0.006) that were greater by more than an order of magnitude. In both groups, the level of HIV RNA in CSF was lower than that found in plasma.

**Table 2:** **Baseline parameter comparisons of low and high groups**

	**Low group (baseline CSF neopterin <21, n = 33)**	**High group (baseline CSF neopterin >21, n = 11)**	***P*** **value**
Age (years)	37 (30 to 46)	36 (28 to 47)	0.87
Days post-transmission	136 (84 to 189)	70 (34 to 191)	0.23
Blood CD4+ T-cells (cells/μL)	601 (444 to 750)	308 (275 to 389)	<0.001
Blood CD8+ T-cells (cells/μL)	993 (692 to 1,269)	1,037 (725 to 1,707)	0.58
Plasma HIV RNA (log10 copies/mL)	4.2 (3.8 to 4.8)	5.3 (4.4 to 5.9)	0.02
CSF HIV RNA (log10 copies/mL)	2.5 (1.7 to 3.2)	3.7 (2.8 to 4.9)	0.006
CSF:plasma HIV RNA ratio (log10)	−1.8 (−2.2 to −1.2)	−1.4 (−2.3 to −0.5)	0.37
CSF WBC count	6 (2.5 to 9.5)	10 (1 to 11)	0.61
Plasma neopterin (nmol/L)	13.9 (10.2 to 18.2)	21.5 (18.3 to 37.5)	0.006
CSF neopterin (nmol/L)	8.5 (5.8 to 11.8)	39.1 (29.2 to 56.6)	<0.001
CSF:plasma neopterin ratio	0.66 (0.42 to 0.8)	1.8 (1.3 to 2.6)	<0.001
CSF:plasma albumin ratio	5.4 (4.3 to 6.4)	7.4 (4.6 to 10.9)	0.06

Unless otherwise stated values are represented as median (interquartile range). CD, cluster of differentiation; CSF, cerebrospinal fluid; WBC, white blood cell count.

**Figure 2:**
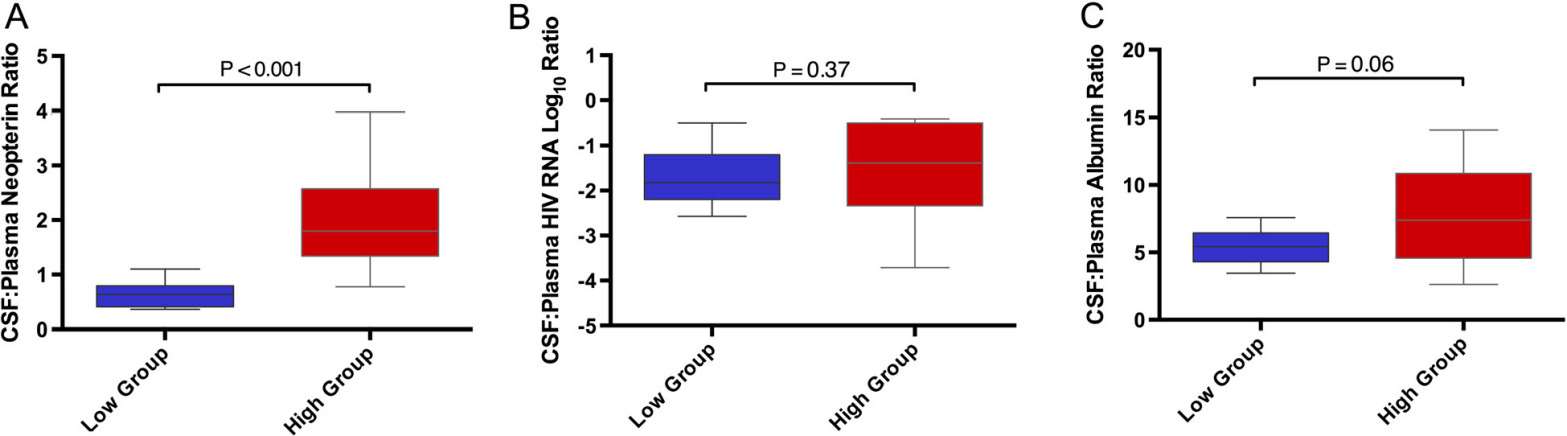
**Cerebrospinal fluid-to-plasma ratios of baseline parameters in low and high groups.** CSF, cerebrospinal fluid. **A)** CSF-to-plasma ratio of neopterin. **B)** CSF-to-plasma ratio of HIV RNA. **C)** CSF-to-plasma ratio of albumin.

Despite a higher CSF-to-plasma neopterin ratio in the high group (Figure 2A, *P* <0.001), the two groups did not differ in the CSF-to-plasma HIV RNA ratio (Figure 2B, *P* = 0.37) or the CSF-to-plasma albumin ratio (Figure 2C, *P* = 0.06). The neopterin-to-HIV RNA ratio was higher in the high group in CSF (*P* <0.001) but not in plasma (*P* = 0.10).

Clinically, two out of 33 subjects in the low group (5.9%) and one out of 11 subjects in the high group (9.1%) had specific neurological seroconversion symptoms (excluding headache). The two neurosymptomatic patients in the low group had Guillain-Barré-like syndrome and meningitis, and the one patient in the high group had meningitis.

### Post-transmission trajectory of activated CD4+ and CD8+ T-cells in blood and cerebrospinal fluid

**Figure 3:**
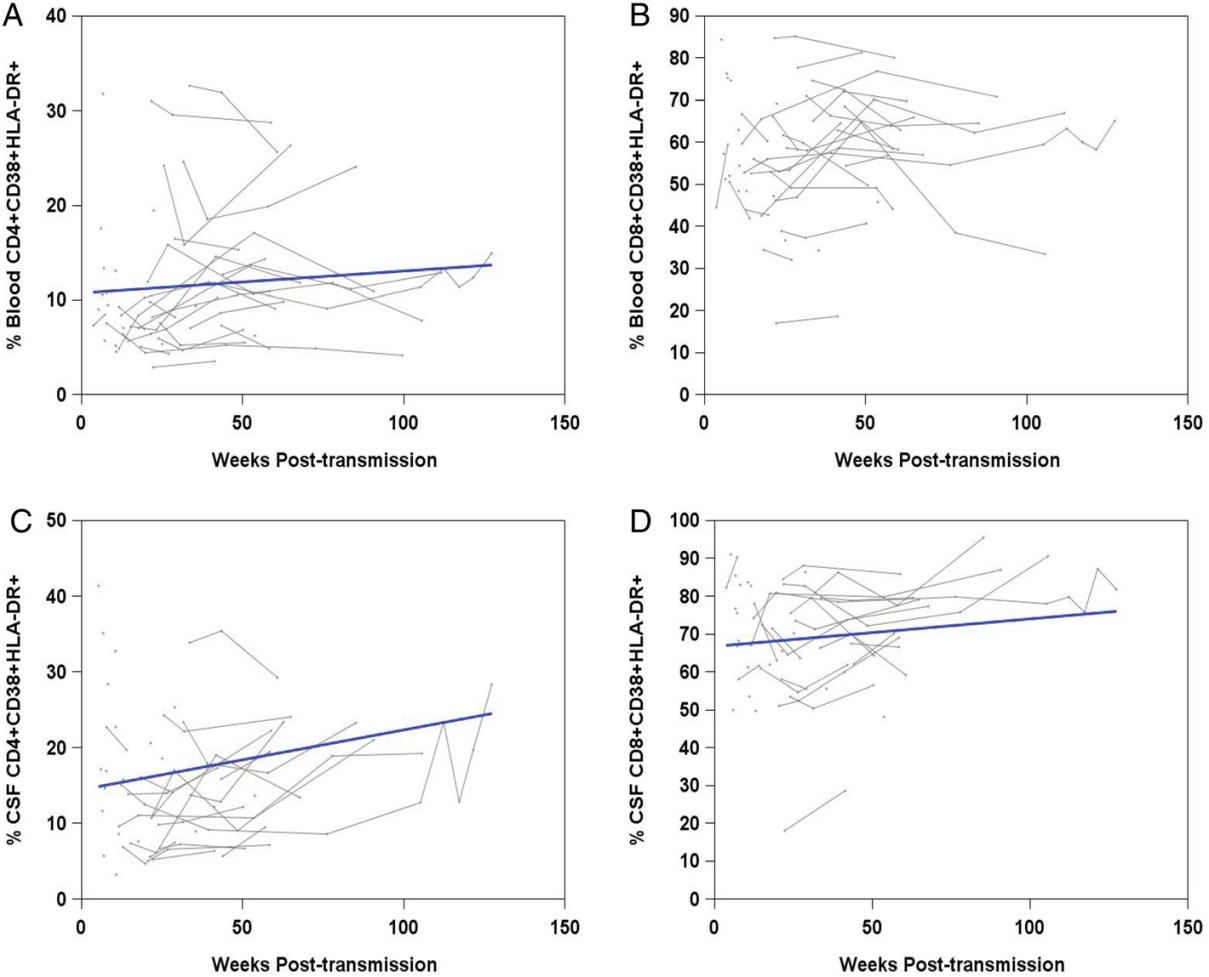
**Trajectories of percentages of activated T-cells in blood and CSF in early HIV-1 infection.** Thin lines represent the trajectories of individual subjects and bold lines represent average slopes obtained from mixed-effects models. **A)** Percentage of CD4+ CD38+ HLA-DR+ cells in blood. **B)** Percentage of CD8+ CD38+ HLA-DR+ cells in blood. No significant trend was observed. **C)** Percentage of CD4+ CD38+ HLA-DR+ cells in CSF. **D)** Percentage of CD8+ CD38+ HLA-DR+ cells in CSF. CD, cluster of differentiation; CSF, cerebrospinal fluid; HLA-DR, human leukocyte antigen-D-related.

Mixed-model analyses of subjects with available flow cytometry data (n = 42) showed an increasing trend in the percentage of activated CD4+ cells in blood at a rate of 0.23 per 10 weeks (*P* = 0.05, Figure 3A). A significant trend for activated CD8+ cells in blood was not found (*P* = 0.54, Figure 3B). In CSF, the percentage of activated CD4+ cells increased at a rate of 0.63 per 10 weeks (*P* = 0.04). We noted an outlier value that had a sharp increase from 8 to 54% from day 26 to 51 post-transmission; re-analysis excluding this outlier yielded a rate of 0.8 per 10 weeks (*P* <0.001, Figure 3C). The percentage of activated CD8+ cells in CSF increased at a rate of 0.73 per 10 weeks (*P* = 0.02, Figure 3D).

## Discussion

In this study, we analyzed the longitudinal levels of CSF neopterin and percentages of activated CD4+ and CD8+ T-cells in blood and CSF, reporting for the first time the natural history of change in these measures of immune activation prior to cART beginning in PHI. HLA-DR and CD38 were used as markers of T-cell activation, and CSF neopterin was used as a soluble marker of CNS inflammation, as it is produced by activated macrophages and astrocytes mainly in response to interferon-gamma. It has been shown that 97.5% of neopterin in CSF is intrathecally produced [24], and it is therefore not simply a spillover from blood, but reflects intrathecal inflammation. Although with any CSF marker there is a concern that it may not accurately reflect processes in the brain, CSF neopterin has been frequently used for the reasons mentioned above as a surrogate biomarker of CNS inflammation and studied as a potential ancillary diagnostic marker for HAD [22,25]. Additionally, CSF neopterin has previously been shown in this PHI cohort to correlate with neurofilament light chain [13], a marker of neuronal injury.

Our analysis showed two trajectories of CSF neopterin beginning in PHI that were dependent on the baseline concentration of CSF neopterin. In the majority of participants, a slow increase was observed, similar to trends in chronic infection [26,27]. Previously unobserved was a downward trend in the quarter of the subjects who had baseline CSF neopterin concentrations above 21 nmol/L, a value obtained through modeling. The median baseline CSF neopterin level in these subjects was more than six-fold greater than the upper limit of normal. Although CSF neopterin decreased over time, the rate was not nearly fast enough for a reduction in CSF neopterin to normal levels within the observed time interval.

To better characterize the groups, we compared them on multiple baseline parameters. In both groups, CSF-to-plasma HIV RNA ratio was lower than the 1 log reduction typically found during chronic infection, as has been previously reported in early HIV infection [11,16]. However, viral load and neopterin concentration in both plasma and CSF were higher in the high group, suggesting a greater degree of systemic and intrathecal inflammation in the initial stage of infection.

Interestingly, despite a lower viral load in CSF compared to plasma in both groups, the concentration of neopterin was higher in CSF than in plasma in the high group, while the converse was true for the low group. Accordingly, a higher CSF-to-plasma neopterin ratio was observed in the high group even though the groups had similar CSF-to-plasma HIV RNA ratios. Further investigation revealed a higher neopterin-to-HIV RNA ratio only in the CSF of the high group, suggesting an amplified intrathecal immune response to the CSF viral load in this group.

Additionally in this study, we analyzed changes in the percentages of activated CD4+ and CD8+ T-cells in blood and CSF, beginning in PHI. A significant trend was not observed for blood CD8+ CD38+ HLA-DR+ cells, a subset that has been associated with the rate of CD4+ cell decline and prognosis in chronic infection [28–30]. However, in CSF, there was an increase in the proportion of activated CD8+ cells. Although associations with HAND in humans are yet unknown, activated CD8+ cells have been shown to produce cytolytic and pro-inflammatory molecules in the brains of macaques with CNS dysfunction from simian immunodeficiency virus (SIV) infection [31]. This observation corroborates the CSF neopterin findings of increasing CNS immune activation beginning in PHI prior to the typical initiation of antiretroviral treatment. The similar upward trajectories for neopterin and activated CD8+ cells in CSF are unsurprising as they were strongly correlated at baseline, consistent with previous reports [20]. These results highlight the utility of CSF neopterin as a marker of CNS inflammation for future studies.

Regarding the percentage of activated CD4+ T-cells in blood, an upward trend was observed in agreement with a previous report of an increase in the density of CD38 on CD4+ cells in blood during early infection [28]. A novel finding in this study was that the percentage of activated CD4+ T-cells also progressively increases in CSF beginning in early infection, which may have implications for the establishment of HIV replication within the CNS compartment, as activated CD4+ cells support viral replication more efficiently compared to resting T-cells [32,33].

## Conclusions

Our longitudinal analyses of both soluble and cellular measures of immune activation indicate that without treatment, CNS inflammation increases during primary HIV-1 infection in most individuals. In a minority of subjects with significantly higher levels of CSF neopterin at baseline, neopterin levels decrease, but only at a slow rate such that elevated levels persist throughout the follow-up period. These results are disconcerting because CNS inflammation and immune activation are considered to be major contributors to neurodegeneration in HIV infection [7,34].

cART dramatically reduces the level of immune activation in blood and CSF [20,35–37], although elevated levels of neopterin and activated T-cells can persist in CSF [38]. Yilmaz *et al*. reported that the set-point of CSF neopterin in subjects on virologically suppressive cART is associated with the pre-ART level of CNS inflammation [39]. Therefore, our observation of a steady increase in CNS inflammation in the majority of subjects highlights the importance of identifying individuals early after infection, and supports prompt initiation of therapy to reduce CNS inflammation in PHI and achieve the lowest possible set-point on cART.

## Abbreviations

cART: combined antiretroviral therapy
CD: cluster of differentiation
CNS: central nervous system
CSF: cerebrospinal fluid
ELISA: enzyme-linked immunosorbent assay
HAD: HIV-associated dementia
HIV: human immunodeficiency virus
HLA-DR: human leukocyte antigen-D-related
HAND: HIV-associated neurocognitive disorder
IQR: interquartile range
PHI: primary HIV infection
SIV: simian immunodeficiency virus
STARHS: serologic testing algorithm for recent HIV seroconversion
ULN: upper limit of normal
WBC: white blood cell
